# Standardized totally endoscopic non-robotic mitral valve repair for degenerative mitral regurgitation: early single-center outcomes in 84 patients

**DOI:** 10.21542/gcsp.2026.30

**Published:** 2026-08-31

**Authors:** Phan Quang Thuan, Pham Tran Viet Chuong, Cao Dang Khang, Nguyen Hoang Dinh

**Affiliations:** 1 Department of Cardiovascular Surgery, University Medical Center, University of Medicine and Pharmacy at Ho Chi Minh City, Ho Chi Minh City, Viet Nam; 2 Department of Cardiovascular and Thoracic Surgery, School of Medicine, University of Medicine and Pharmacy at Ho Chi Minh City, Ho Chi Minh City, Viet Nam

## Abstract

**Objective:** To evaluate early clinical and echocardiographic outcomes of totally endoscopic, non-robotic mitral valve repair for degenerative mitral regurgitation using a standardized DCT approach (double-port; camera and main working port in the same intercostal space; skin incision <3 cm), and to propose operational criteria for “totally endoscopic” mitral surgery.

**Methods:** In this single-center observational study, 84 consecutive patients with severe degenerative mitral regurgitation meeting 2021 ESC/EACTS surgical criteria underwent totally endoscopic non-robotic repair with three-dimensional visualization between July 2023 and January 2026. The technique used a primary skin incision ≤3 cm with no rib spreading in all cases. Clinical and echocardiographic outcomes within 30 days were assessed.

**Results:** Repair was successful in all 84 patients (100%), without conversion to sternotomy or replacement. All underwent ring annuloplasty; neochordal implantation was performed in 69 (82.1%). Mean incision length was 2.9 ± 0.3 cm. Mean cardiopulmonary bypass and cross-clamp times were 163.3 ± 50.5 and 112.1 ± 37.5 minutes, respectively. There was no 30-day mortality. Major complications each occurred in 1 patient (1.2%): re-exploration for bleeding, intracranial hemorrhage, and femoral artery occlusion. Median intensive care unit and hospital stays were 2 (IQR 2–3) and 10 (IQR 9–14) days. Echocardiography showed no residual regurgitation ≥ moderate and preserved ventricular function in all patients.

**Conclusions:** Standardized totally endoscopic non-robotic mitral valve repair using the DCT approach was feasible and reproducible, achieving a 100% repair rate with favorable early outcomes. Given the single-center design and short follow-up, these should be interpreted as feasibility data.

## Introduction

Degenerative mitral regurgitation (DMR) is the most common cause of primary mitral valve disease. Surgical repair remains the treatment of choice and, compared with replacement, is associated with better long-term survival and greater durability of valve function^1^. Over the past two decades, minimally invasive approaches have increasingly supplanted conventional sternotomy, aiming to reduce surgical trauma while preserving the quality and reproducibility of repair.

Robot-assisted mitral valve repair achieves excellent early and mid-term outcomes, largely through enhanced visualization and instrument dexterity. However, its adoption is constrained by high capital and maintenance costs, infrastructure requirements, and limited availability, particularly in low- and middle-income settings^2^. Consequently, interest has grown in totally endoscopic mitral valve repair without robotic assistance as a more accessible and cost-effective alternative^3^.

Several centers have reported favorable results with non-robotic thoracoscopic repair, with outcomes approaching those of robotic series in observational cohorts^4^. Nevertheless, the term “totally endoscopic” is applied inconsistently in the literature, with substantial variation in incision length, port configuration, use of rib spreading, and reliance on direct vision. This absence of a standardized definition hampers comparison of outcomes across studies and limits reproducibility between institutions.

Building on operational criteria for totally endoscopic surgery that our group previously proposed in the settings of ascending aortic replacement and transaortic septal myectomy^6,7^, we adopted a standardized, non-robotic approach for mitral valve repair that preserves technical simplicity and reproducibility. In this study, we report the early clinical and echocardiographic outcomes of this technique in patients undergoing repair for degenerative mitral regurgitation, and we extend these criteria to mitral surgery as a step toward a consistent operational definition of “totally endoscopic” repair.

## Methods

### Study design and patients

This was a single-center retrospective observational study of 84 consecutive patients with severe degenerative mitral regurgitation who underwent surgery between July 2023 and January 2026. All patients met surgical indications according to the 2021 ESC/EACTS Guidelines for the Management of Valvular Heart Disease^5^. The study was approved by the Institutional Review Board of University Medical Center Ho Chi Minh City, and consent for publication of the accompanying video was obtained.

### Definition of “totally endoscopic” repair

**Figure 1.**
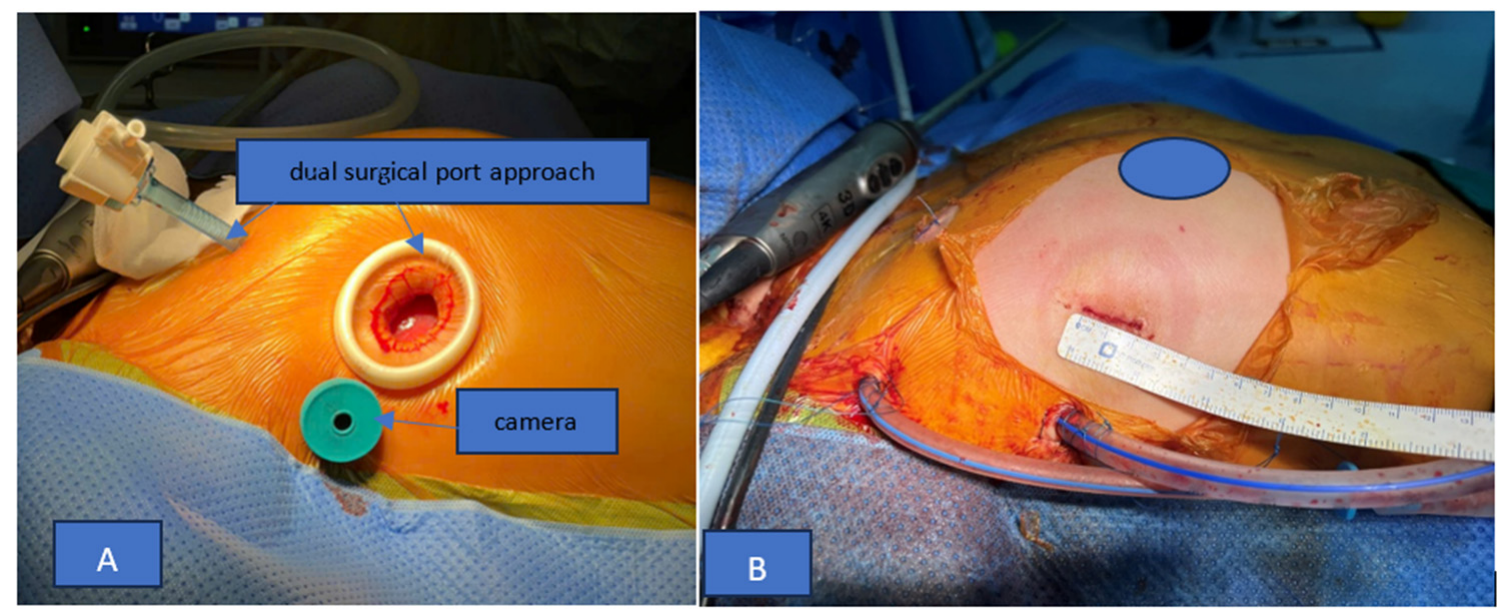
(A) Illustration of the DCT approach, indicated by the arrow; (B) The skin incision measured approximately two cm after skin closure.

All procedures used a standardized totally endoscopic, non-robotic technique with three-dimensional endoscopic visualization. Applying the operational criteria for totally endoscopic surgery previously proposed by our group^6,7^, we defined the approach by: (1) exclusive endoscopic visualization, without direct vision through the incision; (2) no rib spreading, with exposure using a soft-tissue retractor alone; (3) a primary skin incision <3 cm; and (4) thoracoscopic port-based instrumentation for all intracardiac maneuvers. We refer to this configuration as the DCT approach—double-port; camera and main working port in the same intercostal space (ICS); totally endoscopic with a skin incision <3 cm (Figure 1).

### Operative technique

Patients received general anesthesia with double-lumen endotracheal intubation and were positioned supine with right-chest elevation. Cardiopulmonary bypass was established via peripheral cannulation. A right mini-thoracotomy was created in the ICS and fitted with a soft-tissue retractor (Alexis Wound Protector/Retractor, Applied Medical Resources, Rancho Santa Margarita, CA, USA); the endoscopic camera port was placed in the same ICS to achieve coaxial alignment between the endoscope and working instruments. Additional ports were used as required for aortic cross-clamping and cardiac venting. The aorta was cross-clamped and myocardial protection achieved. All intracardiac steps were performed under exclusive endoscopic visualization. Mitral repair techniques were selected according to valve pathology, and repair adequacy was confirmed by intraoperative transesophageal echocardiography (Video S1).

### Outcomes

The primary outcomes were early (30-day) mortality, defined as death from any cause within 30 days of the index operation, and major postoperative morbidity, comprising reoperation for bleeding, acute kidney injury requiring dialysis, low cardiac output syndrome, stroke, thromboembolic events, surgical wound complications, permanent pacemaker implantation, and pneumonia. Transthoracic echocardiography was performed in all patients before discharge to assess mitral valve function. Intensive care unit and hospital length of stay were recorded.

### Statistical analysis

Categorical variables are presented as frequencies and percentages. Continuous variables are expressed as mean ± standard deviation when normally distributed and as median with interquartile range when the distribution was skewed; the distribution of continuous variables was assessed using [the Shapiro–Wilk test/visual inspection]. Analyses were performed using Stata version 17 (StataCorp LLC, College Station, TX, USA).

## Results

Baseline characteristics of the 84 patients are summarized in Table 1. Mean age was 51.8 ± [SD] years, and 61 patients (72.6%) were male. Twelve patients (14.3%) had preoperative atrial fibrillation. Eighteen patients (21.4%) had Barlow disease, and 26 (31.0%) met the criteria of Anyanwu and colleagues for complex mitral valve pathology^8^.

**Table 1:** Baseline patient profiles (*n* = 84).

**Characteristics**	**n (%) or mean ± SD**	
**Demography**	
Male	61 (72.6)	
Age (years), median (interquartile range)	53 (43–60)	
Body surface area (m2)	1.7 ± 0.2	
Body max index	22.7 ± 3.0	
**Clinical data**	
Atrial fibrillation	12 (14.3)	
Hypertension	31 (36.9)	
Diabetes mellitus	4 (4.8)	
Hypercholesterolaemia	19 (22.6)	
History of stroke	
Chronic obstructive pulmonary disease	1 (1.2)	
Chronic kidney disease	17 (20.2)	
Dialysis	1 (1.2)	
Left Main Disease	0 (0)	
Coronary Artery Disease (LAD)	3 (3.6)	
Coronary Artery Disease (Circumflex)	2 (2.4)	
Right Coronary Artery Disease	4 (4.8)	
Previous sternotomy	0 (0)	
NYHA III or IV	20 (27.4)	
No symtom	11 (13.1)	
Euroscore II (mean, min –max)	1.1 (0.6 –3.4)	
**Echocardiographic data**	
LV ejection fraction (%)	62.5 ± 4.0	
LV systolic dimension (mm)	35.8 ± 4.7	
LV diastolic dimension (mm)	46.6 ± 12.1	
LA dimension (mm)	50.7 ± 10.0	
Moderate tricuspid regurgitation	26 (31.0)	
Severe tricuspid regurgitation	4 (4.8)	
FAC (%)	39.9 ± 7.6	
PAPs	37.8 ± 16.3	
Right ventricle failure	5 (6)	
Severe degenerative mitral regurgitation	84 (100.0)	
Barlow	18 (21.4)	
Complex mitral valve pathology	26 (31.0)	

**Notes.**

LV left ventricular LA Left atrial LAD Left Anterior Descending FAC Fractional Area Change PAPs systolic pulmonary artery pressure NYHA New York Heart Association

Operative data are summarized in Table 2. Mitral valve repair was achieved in all patients (84/84, 100%), with no conversion to valve replacement or sternotomy. Ring annuloplasty was performed in all cases. Repair techniques included neochordal implantation in 69 patients (82.1%), the Roman arch technique described by Kofidis and colleagues in 6 (7.1%)^9^, and triangular resection in 2 (2.4%). Concomitant procedures comprised tricuspid valve repair in 9 patients (10.7%), a maze procedure in 2 (2.4%), and combined tricuspid repair with maze in 1 (1.2%). Peripheral cannulation was used to establish cardiopulmonary bypass in 38 patients (45.2%). Mean cardiopulmonary bypass and aortic cross-clamp times were 147.7 ± 51.5 and 127.8 ± 49.4 min, respectively. Mean primary skin incision length was 2.9 ± 0.3 cm.

**Table 2:** Operative characteristics (*n* = 84).

**Characteristics**	**n (%) or mean ± SD**
**Leaflet manipulation**	
Anterior leaflet	10 (11.9)
Posterior leaflet	30 (35.7)
Bileaflet	44 (52.4)
Roman Arch	6 (7.1)
**Mitral repair procedure**	
Ring annuloplasty	84 (100.0)
Neochordae implantation	69 (82.1)
Number of neochordae implantation	1.2 ± 0.6
Commissuroplasty	35 (41.7)
Leaflet plication	39 (46.4)
Indentation closure	26 (31.0)
Triangular/quadrangular resection	2 (2.4)
Edge-to-edge technique	13 (15.5)
**Concomitant procedures**	
Tricuspid annuloplasty	9 (10.7)
Atrial fibrillation ablation (Maze procedure)	2 (2.4)
Tricuspid annuloplasty and Maze procedure	1 (1.2)
Cardiopulmonary bypass	
Cardiopulmonary bypass time (min)	163.3 ± 50.5
Percutaneous cardiopulmonary bypass	38 (45.2)
Aortic cross-clamping time (min)	112.1 ± 37.5
primary skin incision length (cm)	2.9 ± 0.3

There were no 30-day deaths. Predischarge transthoracic echocardiography, performed in all patients, demonstrated no residual mitral regurgitation of moderate or greater severity (Tables 3 and 4). Major postoperative complications each occurred in 1 patient (1.2%): thoracoscopic re-exploration for bleeding; anticoagulation-related intracranial hemorrhage managed with decompressive craniotomy; and femoral artery occlusion following percutaneous (ProGlide) closure, requiring [specify: surgical thrombectomy/endovascular repair]. All patients survived to discharge. Median intensive care unit and postoperative hospital stays were 2 days (IQR 2–3) and 10 days (IQR 9–14), respectively.

**Table 3:** Postoperative outcomes and complications (*n* = 84).

**Characteristics**	**n (%) or mean ± SD**
Early death (<30 days)	0 (0.0)
Ventilation time (hours)	7 ± 5.6
ICU stay (median days, interquartile range)	2 (2–3)
Hospital stay (median days, interquartile range)	10 (9–14)
**Complications**	
Reoperations for bleeding	1 (1.2)
Sternotomy conversion	0 (0.0)
Femoral artery occlusion related to percutaneous closure	1 (1.2)
Low cardiac output syndrome	0 (0.0)
Pneumonia	2 (2.4)
Permanent pacemaker insertion	2 (2.4)
Thoracotomy wound	0 (0.0)
Stroke (hemorrhagic or ischemic)	3 (3.6)
Intracranial hemorrhage related to anticoagulation and underwent decompressive craniotomy	1 (1.2)
Thromboembolic event	0 (0.0)
Acute kidney injury requiring dialysis	1 (1.2)
Urinary tract infection	0 (0.0)
Sepsis	0 (0.0)
Reoperation for mitral regurgitation	0 (0.0)

**Table 4:** Early postoperative echocardiographic findings (*n* = 84).

**Characteristics**	**n (%) or mean ± SD**
Days after the operation	2.4 ± 2.7
**Mitral regurgitation**	
None or trace	33 (39.3)
Mild	51 (60.7)
Moderate or severe	0 (0.0)
**Tricuspid regurgitation**	
None or trace	38 (45.2)
Mild	40 (47.6)
Moderate or severe	6 (7.1)
LV ejection fraction (%)	54.8 ± 5.6
LV systolic dimension (mm)	34.3 ± 5.8
LV diastolic dimension (mm)	48.4 ± 6.5
LA dimension (mm)	35.8 ± 5.8
FAC (%)	40.0 ± 7.1
PAPs	29.9 ± 9.2

**Notes.**

LV left ventricular LA Left atrial FAC Fractional Area Change PAPs systolic pulmonary artery pressure

## Discussion

In this single-center experience of 84 consecutive patients with severe degenerative mitral regurgitation, a standardized totally endoscopic, non-robotic repair strategy using three-dimensional visualization achieved a 100% repair rate with no conversion to sternotomy or replacement, despite a substantial proportion of challenging anatomy (Barlow disease, 21.4%; complex pathology, 31.0%). These findings support the feasibility of a non-robotic endoscopic platform for reliable early results when a reproducible workflow is applied.

Comparison of “totally endoscopic” outcomes across the literature is limited by the absence of a uniform definition. Reported approaches differ substantially between centers, particularly in the weight given to incision length versus the functional principle of operating through ports under exclusive endoscopic visualization, and this ambiguity contributes to the heterogeneity of series labelled “totally endoscopic”^4^. The DCT configuration is intended to specify one reproducible, endoscope-dominant setup—coaxial instrument geometry through a double-port arrangement in a single intercostal space, without enlargement of the primary access^6,7^. The consistently small primary incision (2.9 ± 0.3 cm) provides an objective marker that repair was completed without escalation to a mini-thoracotomy working window. We therefore position the DCT approach not as a competing definition of “totally endoscopic” surgery, but as an explicit, measurable set of criteria that others can apply or contest.

Early outcomes were encouraging. There was no early mortality, and recovery was rapid, with a median intensive care unit stay of 2 days (IQR 2–3) and hospital stay of 10 days (IQR 9–14). Major complications were uncommon, each occurring in 1 patient (1.2%): reoperation for bleeding, acute kidney injury requiring dialysis, and femoral artery occlusion related to percutaneous closure. Neurologic events occurred in 3 patients (3.6%). No patient required reoperation for recurrent mitral regurgitation before discharge.

Mean cardiopulmonary bypass and cross-clamp times were 163.3 ± 50.5 and 112.1 ± 37.5 minutes, respectively. The range of repair maneuvers—neochordal implantation (82.1%), leaflet plication (46.4%), commissuroplasty (41.7%), indentation closure (31.0%), and edge-to-edge repair (15.5%)—indicates that endoscopic exposure and instrument handling permitted a complete repertoire rather than technique-limited repairs. This is relevant because the perceived constraint of non-robotic totally endoscopic surgery is the ability to address bileaflet or complex lesions, yet bileaflet involvement was present in 52.4% of this cohort and repair was achieved in all cases. These operative times and this case complexity fall within the range reported for contemporary robotic mitral repair^2,10^, although the single-arm design precludes direct comparison.

Percutaneous peripheral cannulation was used in 45.2% of cases, a perfusion strategy that can reduce groin dissection and streamline setup. The single femoral artery occlusion related to percutaneous closure (1 of 38 patients undergoing percutaneous access, 2.6%) underscores the need for careful cannulation planning, ultrasound guidance, meticulous closure, and postoperative limb surveillance in programs adopting percutaneous access.

Echocardiography within 30 days showed no residual mitral regurgitation of moderate or greater severity; regurgitation was absent or trace in 39.3% of patients and mild in 60.7%. Left ventricular ejection fraction was preserved (54.8 ±5.6%) with satisfactory chamber dimensions, and right-sided parameters were largely normal, with preserved right ventricular function (fractional area change 40.0 ±7.1%) and no more than mild tricuspid regurgitation in most patients. These findings indicate effective early valve competence. Longer follow-up will be required to establish repair durability. Our early results are consistent with those of other non-robotic totally endoscopic series^11,12^.

## Limitations and future directions

This study has several limitations. It was a retrospective, single-center analysis of a modest cohort operated by a single experienced team, which limits generalizability and leaves the findings subject to selection bias; the 100% repair rate in particular should be read in that light. Outcomes were confined to the first 30 postoperative days, so the analysis cannot address repair durability, freedom from reoperation, or the clinical significance of the mild residual regurgitation observed in the majority of patients—all of which require longer echocardiographic follow-up.

The single-arm design precludes direct comparison with robotic or mini-thoracotomy approaches; the comparisons drawn with published series are descriptive only. Finally, because “totally endoscopic” is defined inconsistently across reports, cross-center comparison remains imperfect until the field converges on standardized reporting elements—incision metrics, rib spreading, camera and port configuration, and explicit conversion thresholds.

Future work should include longer-term clinical and echocardiographic follow-up, risk-adjusted comparison with robotic and mini-thoracotomy cohorts, and evaluation of the DCT criteria by other groups. We offer these criteria as one contribution toward the consensus terminology the field will need for meaningful benchmarking, rather than as a settled definition.

## Conclusion

Totally endoscopic non-robotic mitral valve repair using a standardized DCT approach was feasible and reproducible in this single-center series, achieving a high repair rate without conversion to sternotomy or valve replacement and no residual mitral regurgitation of moderate or greater severity at early follow-up. These findings support the early safety and technical adequacy of the approach in patients with degenerative mitral regurgitation. Confirmation of durability will require longer follow-up and comparison with established approaches. We offer the DCT criteria as a reproducible, measurable definition of the totally endoscopic setup that other centers can apply and test, as a step toward the standardized reporting the field currently lacks.
